# Allergic Rhinitis: What Do We Know About Allergen-Specific Immunotherapy?

**DOI:** 10.3389/falgy.2021.747323

**Published:** 2021-10-28

**Authors:** Tadech Boonpiyathad, Mongkol Lao-Araya, Chirawat Chiewchalermsri, Sasipa Sangkanjanavanich, Hideaki Morita

**Affiliations:** ^1^Department of Medicine, Phramongkutklao Hospital, Bangkok, Thailand; ^2^Faculty of Medicine, Department of Pediatrics, Chiang Mai University, Chiang Mai, Thailand; ^3^Department of Medicine, Panyananthaphikkhu Chonprathan Medical Center, Srinakharinwirot University, Nonthaburi, Thailand; ^4^Faculty of Medicine Ramathibodi Hospital, Department of Medicine, Mahidol University, Bangkok, Thailand; ^5^Department of Allergy and Clinical Immunology, National Research Institute for Child Health and Development, Tokyo, Japan; ^6^Allergy Center, National Center for Child Health and Development, Tokyo, Japan

**Keywords:** allergic, rhinitis, immunotherapy, allergen-specific, immune tolerance

## Abstract

Allergic rhinitis (AR) is an IgE-mediated disease that is characterized by Th2 joint inflammation. Allergen-specific immunotherapy (AIT) is indicated for AR when symptoms remain uncontrolled despite medication and allergen avoidance. AIT is considered to have been effective if it alleviated allergic symptoms, decreased medication use, improved the quality of life even after treatment cessation, and prevented the progression of AR to asthma and the onset of new sensitization. AIT can be administered subcutaneously or sublingually, and novel routes are still being developed, such as intra-lymphatically and epicutaneously. AIT aims at inducing allergen tolerance through modification of innate and adaptive immunologic responses. The main mechanism of AIT is control of type 2 inflammatory cells through induction of various functional regulatory cells such as regulatory T cells (Tregs), follicular T cells (Tfr), B cells (Bregs), dendritic cells (DCregs), innate lymphoid cells (IL-10^+^ ILCs), and natural killer cells (NKregs). However, AIT has a number of disadvantages: the long treatment period required to achieve greater efficacy, high cost, systemic allergic reactions, and the absence of a biomarker for predicting treatment responders. Currently, adjunctive therapies, vaccine adjuvants, and novel vaccine technologies are being studied to overcome the problems associated with AIT. This review presents an updated overview of AIT, with a special focus on AR.

## Introduction

**Figure 1:**
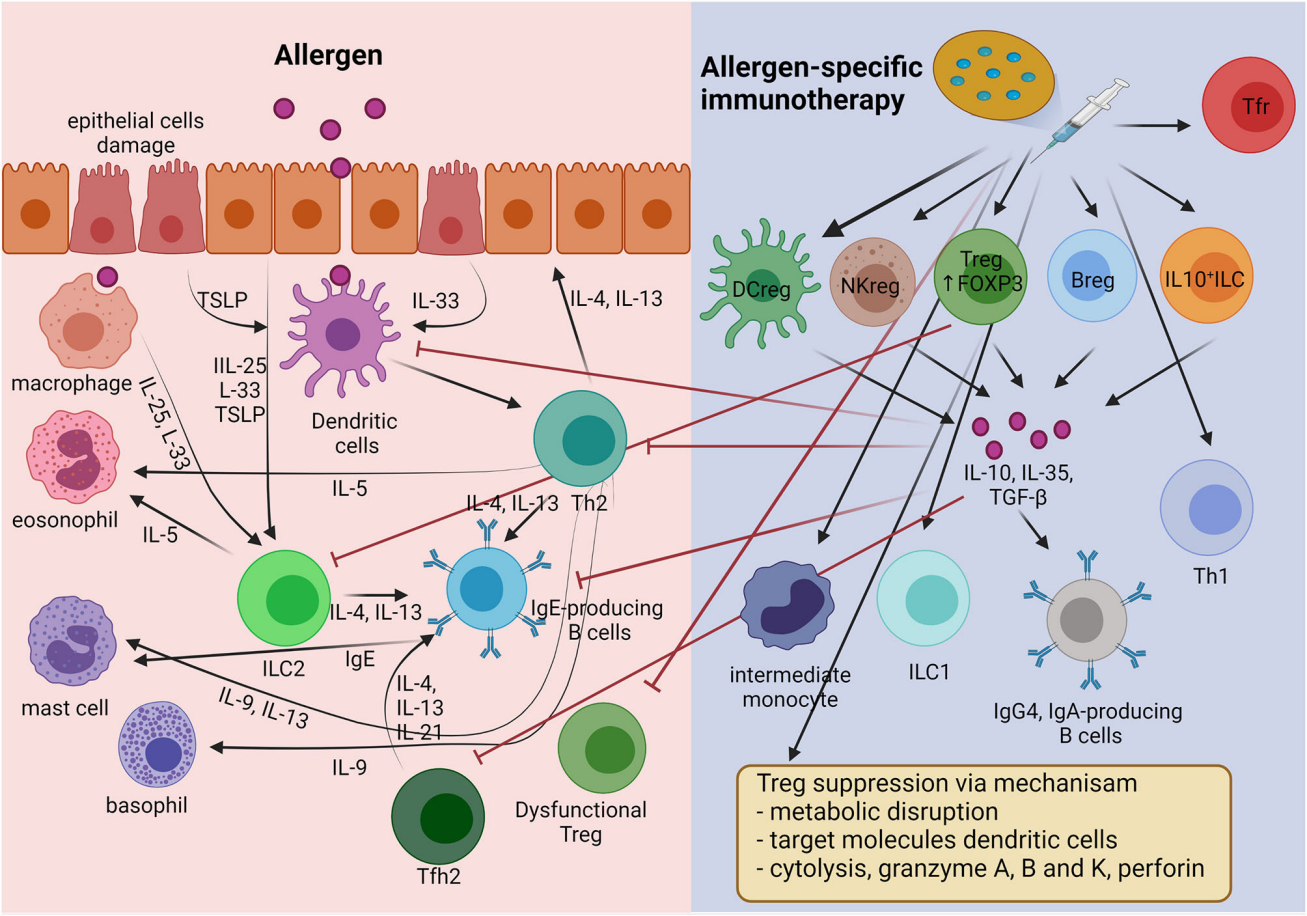
The mechanism of immune tolerance to allergen induces by allergen-specific immunotherapy (AIT). AIT principally induces regulatory cells including Treg, Breg, Tfr, DCreg, NKreg, and IL-10^+^ ILC cells. Treg cells apply four main mechanisms for suppressing inflammatory cells (inhibitory cytokines, cytolysis, metabolic disruption, and targeting DCs). In addition, the regulatory cells produce IL-10 to suppress the type 2 inflammatory cells involved in allergic inflammation, such as Th2, Tfh2, IgE-producing B cells, and ILC2s. Moreover, AIT induces allergen-specific immunoglobulin class-switch, promoting IgG4 and IgA.

Allergic rhinitis is a common upper airway disease. Its prevalence varies around the world. A good epidemiologic study reported that 20 to 30% of adults and up to 40% of children are affected (1). We recognize that allergic rhinitis (AR) has significant effects on the quality of life, sleep, and performance at work and school of patients. AR is not only a disease of the upper airway. It may also lead to inflammatory processes in the lower airways, which is supported by the fact that rhinitis and asthma frequently coexist (2). Allergies are characterized by dysregulated type 2 immunity and epithelial barriers that have increased concentrations of allergen-specific immunoglobulin (Ig) E (3, 4). Type 2 immune responses involve T helper (Th) 2 cells, IgE-producing B cells, group 2 innate lymphoid cells (ILC2s), and small fractions of interleukin (IL)-4-producing natural killer (NK) cells and NK-T cells, basophils, eosinophils, mast cells, and their cytokines (5). Emerging evidence suggests that follicular helper T (Tfh) cells, rather than Th2 cells, play a crucial role in controlling IgE production (6). Upregulation of Tfh cell activities, including a skewing toward type 2 Tfh cells and IL-13-producing Tfh phenotypes, and defects in follicular regulatory T cells (Tfr) have been recognized in patients with allergic diseases (6). Moreover, there is a complex network among type 2 cytokines (IL-4, IL-5, IL-9, and IL-13) which are secreted mainly from type 2 immune cells, and alarmins [IL-25, IL-33, and thymic stromal lymphopoietin (TSLP)] which are released from tissue cells, particularly epithelial cells (Figure 1).

Basic AR treatment consists of allergen avoidance, use of medications that provide symptomatic relief, anti-inflammatory therapies, and allergen-specific immunotherapy (AIT). At present, AIT is only disease-modifying, and it is aimed at improving allergen tolerance. AIT also changes the allergic immune response to one of immune tolerance, as in healthy individuals (7). AIT uses general mechanisms of immune tolerance to allergens to normalize allergen-specific T and B cells, regulation of IgE and IgG production, and modification of mast cells, basophil activation thresholds, and the phenotype of dendritic cells (DCs) (8). The main goals are maintaining regulatory T cells (Tregs), regulatory B cells (Bregs), and various other regulatory cells in order to suppress type 2 immune responses and allergic inflammation (Figure 1) (9). AIT showed efficacy in selected AR patients with HDM and birch or grass-pollen sensitization (10, 11). Substantial evidence supports the effectiveness of AIT for AR in reducing the symptoms and medication requirements, and its safety and cost-effectiveness (12). AIT applied in the early stage of allergic disease had an excellent preventive effect on disease progression to asthma, especially in young children (13). However, significant limiting factors for AIT were the long duration of treatment, cost, poor patient compliance, and severe life-threatening adverse reactions to the treatment (14). It is hoped that these disadvantages can be mitigated by developing non-allergenic, highly immunogenic allergen extracts, combined usage with novel adjuvant molecules, and new administration routes. Here, we review our current knowledge regarding AIT for AR. In addition, we update relevant topics on the use of AIT in AR that can help physicians in daily practice.

## The Cellular Immune Response Following AIT

Since AIT acts in an antigen-specific manner, modulation of antigen-specific immune cells, including T and B cells, was thought to be its primary mode of action. However, recent findings suggest that AIT also modulates non-antigen-specific immune cells, including ILCs, monocytes/macrophages, NKs, and DCs. These effects may also contribute to the improvement of symptoms after AIT.

### T Cells

AIT induces FOXP3^+^ and IL-10^+^ Treg cells (Tregs), which prevent and inhibit allergic inflammation by expressing their immunosuppressive functions at different levels (15). There are 4 types of the suppressive mechanism used by Tregs: (1) via suppressive cytokines, IL-10, IL-35, and transforming growth factor-β (TGF-β) secretion, (2) disruption of metabolic pathways via CD25, cAMP, adenosine receptor 2, histamine receptor 2 (HR2), CD39, and CD73, (3) suppression of DC activation by membrane-bound molecules, programmed death 1 (PD-1), and cytotoxic T lymphocyte antigen 4 (CTLA-4), and (4) cytolysis (granzymes A, B, and K) (16, 17). IL-10–producing Tregs suppress Th2 type immune responses (IL-4, IL-5, IL-9, and IL-13) and IL-17–producing Th cells (18, 19). Moreover, AIT can inhibit CD45RB^low^CD27^−^CRTH2^+^CD161^+^CD49d^+^ T cells (Th2A) and IL-21^+^ Tfh cells (20, 21). On the other hand, AIT promotes IL-22– and interferon-γ (IFN-γ)–producing Th cells (22, 23). AIT also involves upregulation of activated Tregs (FOXP3^+^Helios^+^CD25^+^CD127^−^) and downregulation of dysfunctional Tregs (ILT3^+^CD25^+^ and FOXP3^+^SATB1^+^) (23, 24). Recent studies revealed that AIT improved dysfunctional Tfr (CD45RA^low^CXCR5^high^FOXP3^+^) and reduced type 2 Tfh cells that contributed to aberrant IgE production (25–27).

### B Cells

Patients responding to AIT are characterized by the increase of IgA, IgD, IgG2 and IgG4-positive allergen-specific B cells, plasmablasts, and IL-10 or IL-1RA-positive Bregs (28–30). IL-10 suppresses IgE production and augments IgG4-producing class-switched B cells (31). IgG4 blocks IgE antibodies by mopping up free allergen, and IgE fails to trigger Fc receptors. Moreover, IgG4 prevents mast–cell activation through FcγIII. AIT also enhanced local allergen-specific IgA1 and IgA2 in patients with grass–pollen allergy (32, 33). Thus, secretory IgA provides protection by blocking allergens absorbed into the mucosa. AIT induces allergen-specific IgD, and a recent study demonstrated that IgD constrains IgE-mediated basophil degranulation (34). Interestingly, a study in patients with grass-pollen subcutaneous immunotherapy (SCIT) found that AIT could induce nasal IgG4 levels, and blocking activity correlated with the clinical response (35).

### Innate Lymphoid Cells

Innate lymphoid cells were recently identified as innate-type immune cells with no antigen receptors, meaning that they are not directly activated by antigens (36). ILCs were activated by various cytokines, neuropeptides, and lipid mediators produced by surrounding cells (37). ILCs were initially divided into three different subsets that resemble Th cell subsets based on the transcription factors and cytokines they produced. Among them, ILC2s resembling Th2 cells were involved in the pathophysiology of various allergic diseases, including asthma and AR, through the production of type 2 cytokines (38). Indeed, the frequency of ILC2s in peripheral blood of seasonal AR patients was increased during the season compared to healthy individuals (39, 40). Local allergen provocation in patients with AR induced accumulation of ILC2s in the nasal tissue, accompanied by increased levels of prostaglandin D2 (PGD2), and IL-5 in the nasal lining fluid (41). These findings suggested that allergen exposure indirectly induces migration and activation of ILC2s through PGD2 synthesis by activated mast cells. AIT reduced the seasonal increase in ILC2s in peripheral blood of patients with seasonal AR (39). Likewise, AIT reduced the frequency of ILC2s in peripheral blood of patients with house dust mite (HDM) AR (42, 43).

Recently, ILCs that produce IL-10 were identified in tissues of both humans (44–46) and mice (44, 47–50). Such cells were rarely detected in the tissues of both humans and mice at a steady state (44, 50). However, they were increased in tissues with type 2 inflammation, such as the nasal tissues of patients with chronic rhinosinusitis with nasal polyps (44), and in the lungs of a murine asthma model (44, 47–50). IL-10-producing ILCs were shown to be converted from ILC2s upon IL-33 and retinoic acid stimulation *in vitro* (44, 46, 47), and they are now considered to be inducible cell types rather than residential cell types. Intriguingly, AIT induced IL-10-producing ILCs in the peripheral blood of patients with HDM (45) and grass-pollen AR (46), and the frequency of those cells correlated with the improvement in the symptom score. These findings suggest that induction of IL-10-producing ILCs is also involved in the mechanisms of AIT. Furthermore, IL-10-producing ILCs were shown to suppress proliferation of ILC2s and T cells through IL-10 and to protect against disruption of epithelial barrier integrity by allergen exposure (44, 46). In murine asthma models, IL-10-producing ILCs reportedly exhibited an exhausted-like phenotype with reduced capacity for type 2 cytokine production (48, 51). However, the mechanisms underlying the induction of AIT of IL-10-producing ILCs remain unclear.

### Dendritic Cells

Dendritic cells are crucial antigen-presenting cells that direct immune responses toward either inducing inflammation or tolerance and are considered to be heterogeneous, both phenotypically and functionally (52). Among them, tolerogenic DCs (tDCs) induce tolerance through various mechanisms, including induction of Tregs (53). Since tDCs are also heterogeneous and may exhibit different phenotypes depending on the organ, the characteristics of tDCs that may be induced by AIT remain unclear. However, some markers related to tDCs, including *complement component 1* and *stabilin*, were upregulated in peripheral blood mononuclear cells (PBMCs) from grass-pollen allergy patients after 4 months of AIT (54), suggesting that induction of tDCs may play a role in AIT. Regulation of DC activation is a key mediated immune response to allergens. Therefore, patients with allergic disease display a tendency to produce fewer tolerogenic IL-10-producing DCs (55). Furthermore, AIT enhanced regulatory dendritic cells (DCregs) and type 1 DCs (DC1s), while decreasing DC2s and DC17s in responder AIT patients (56). Plasmacytoid DCs (pDCs), which play a crucial role in immunity against viral infections, were suggested to be involved in the mechanisms of AIT. Eljaszewicz et al. reported an increase in pDCs and CD141^+^ myeloid DCs in individuals with allergies (43). In contrast, the number of CD1c^+^ myeloid DCs in patients with AR decreased during the first year of AIT (43). Also, pDCs in peripheral blood were found to be decreased in number after AIT (57, 58).

### Macrophages

Macrophages are heterogenous phagocytic cells that play a vital role in innate immunity and are significant contributors to the adaptive immune system. Macrophages activated by Th1 cells are identified as M1 macrophages, while those activated by IL-4 and IL-13 are named alternatively activated macrophages (AAMs) or M2 cells (59). M2 macrophages can produce IL-4 and IL-13, and IL-10 and TGF-β in response to specific stimulators. M2a cells activate Th2 cells via IL-4 and IL-13 production mediated by C-C Motif chemokine ligand (CCL) 17 and mannose receptor C-Type 1 (MRC1), leading to the development of allergic asthma (60). M2b cells activate Tregs via IL-10 and TGF-β production mediated by CCL24 and MRC1, leading to allergic tolerance and deceased inflammation (60). However, the roles of M2 macrophages in AIT need to be further investigated.

### Monocytes

Circulating monocytes are also known to be heterogeneous and include 3 distinct subsets: classical monocytes (CD14^++^CD16^−^), intermediate monocytes (CD14^++^CD16^+^), and non-classical monocytes (CD14^+^CD16^++^). Non-classical monocytes are considered to be proinflammatory cells that produce large amounts of TNF-α. The frequency of non-classical monocytes in peripheral blood was decreased after 3 months of AIT and was more pronounced after 6 months. On the other hand, intermediate monocytes, thought to have anti-inflammatory properties, were increased after 1 year of AIT (61). Sousa et al. also found that AIT enhanced circulating CD16^+^ monocytes (57).

### NK Cells

Natural killer cells can differentiate into 2 distinct functional subsets: NK1 or NK2 cells, which are analogous to the T-cell subsets Th1 or Th2 (62). Moreover, TGF-β and IL-10-secreting NKreg cells might have a role in the immune regulation of allergic inflammation. For example, IL-10-producing NKreg cells significantly suppressed both allergen or antigen-induced T-cell proliferation, IL-13 and IFN-γ-secreting T cells, and reduced IgE production (62, 63). However, a recent study observed no changes in the frequency of NK cells in patients undergoing AIT (43).

## Administration Routes

### Subcutaneous

Until recently, subcutaneous delivery (SCIT) was the standard administration route for AIT (64). The conventional schedule for SCIT using allergen extracts consists of dose build-up by once-weekly injection, followed by maintenance dose injections at 4–8-week intervals, continued for at least 3–5 years (65). The build-up phase can be shortened by following cluster or rush protocols to help the patients reach maintenance (66). In the cluster protocol, multiple injections are given on non-consecutive days. In contrast, in the rush protocol, multiple injections are given on consecutive days, reaching the maintenance phase in few days, but this increases the risk of anaphylaxis (67). Therefore, the accelerated protocols should be applied only in specialized centers.

### Sublingual

**Figure 2:**
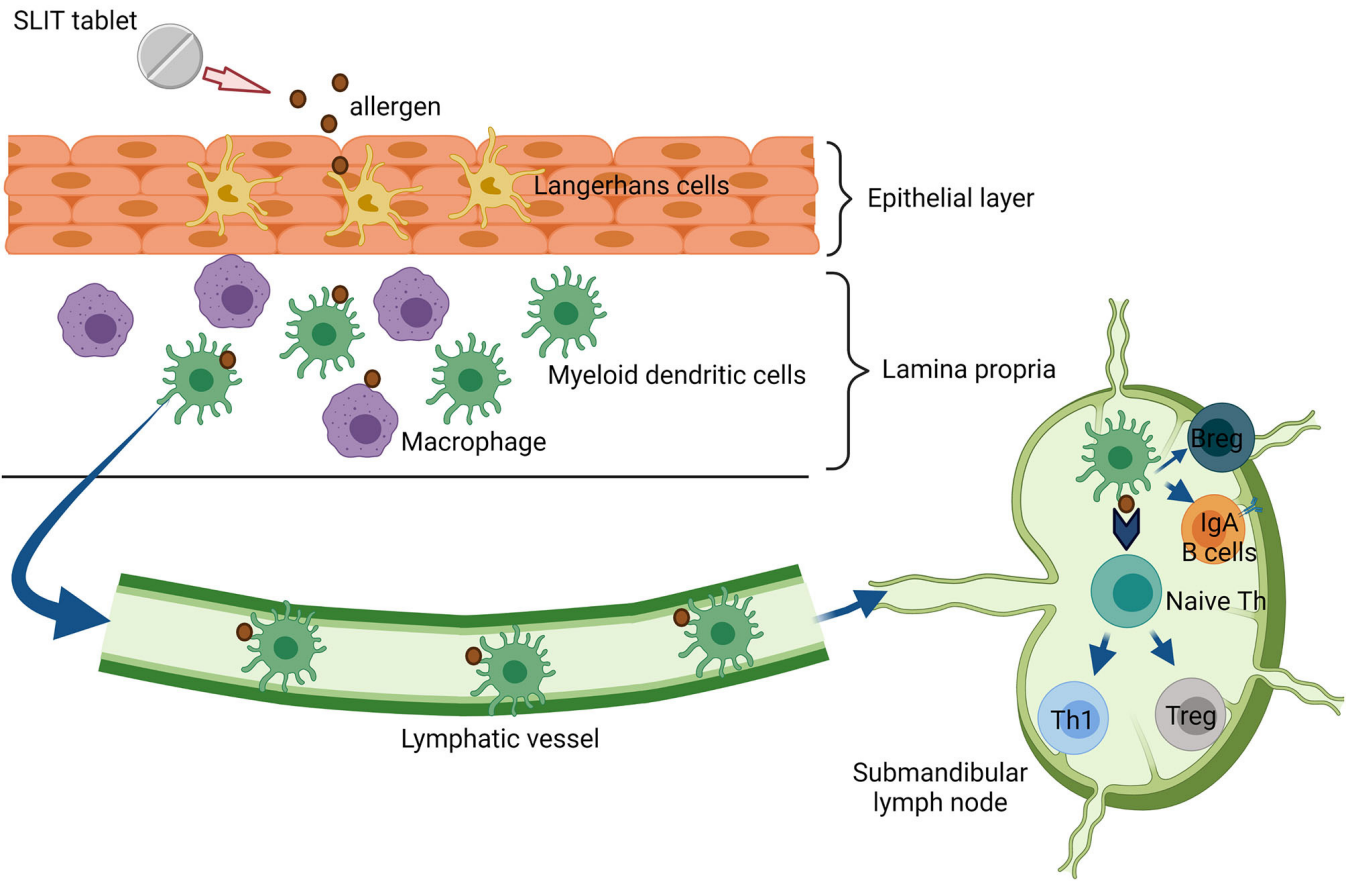
Immunologic mechanism of sublingual immunotherapy (SLIT). Substantial amounts of allergens from SLIT-tablet are capture by Langerhans cells in the epithelial layer. Next, the allergen is processed and migrated transmucosal by myeloid dendritic cells (mDCs) to draining lymph nodes. In the lymph node, these cells interact with T and B cell priming to regulate immune tolerance.

Sublingual immunotherapy involves administering allergens under the tongue, generally daily. Sublingual immunotherapy (SLIT) is administered *via* liquid drops or as freeze-dried, lyophilized, or film-coated tablets. SLIT tablets contain a single allergen, whereas SLIT drops often contain multiple allergens for the treatment of poly-sensitization (68). At present, SLIT is widely used to treat HDM, and grass and tree-pollen allergies. Also, SLIT can be safely and effectively performed at home, and it does not require a build-up phase (69). The oral mucosa and regional lymph nodes form an elaborate immunological network, which is an essential prerequisite for SLIT. The network includes local antigen-presenting cells (APCs), such as Langerhans cells in the epithelium, and oral dendritic cells (DCs) with the CD103C^−^CD11b^+^ phenotype and macrophages in the lamina propia (Figure 2) (70). Oral DCs transport sublingual antigens to the submandibular lymph nodes and induce antigen-specific Tregs. In addition, SLIT induces mucosal and serum-specific-IgA responses, which may contribute significantly to tolerance induction (32, 71). A clear difference between SCIT and SLIT is the effective dosing range of allergen management. SCIT uses a narrow effective dosing range of 5–25 μg of allergen per injection for many allergens, whereas SLIT requires at least 50–100 times more allergen than SCIT to achieve a similar level of efficacy (72). Therefore, long-term compliance with SLIT might be a concern. However, a recent study in Denmark reported similar 1-year compliance of ~50% with both SCIT and SLIT (73).

### Intra-Lymphatic

Intra-lymphatic immunotherapy (ILIT) is the direct intra-lymphatic injection of allergens. ILIT improves the efficiency of AIT by reducing the number of treatment applications and the treatment duration, achieving good compliance and fast symptom alleviation, and showing exemplary safety (74, 75). ILIT requires only three ultrasound-guided injections of a low allergen dose into the inguinal lymph nodes at 1-month intervals (76). The cumulative allergen dose can be reduced 1,000-fold compared to SCIT (77). The disadvantage of ILIT is the requirement for experienced staff for injection under ultrasound guidance.

### Epicutaneous

Epicutaneous immunotherapy is a novel therapy that is currently being investigated. Epicutaneous immunotherapy (EPIT) delivers allergens via repeated applications to the skin and APCs in the superficial skin layers (78). Innovative epidermal allergen powder delivery technologies include electronic spreading, ablative fractional laser, and microneedle arrays (79). By targeting epidermal Langerhans cells, but not mast cells or the vasculature, EPIT can reduce both local and systemic adverse effects (80). The following advantages have been noted for EPIT: (1) a high safety profile due to allergen application into the non-vascularized epidermis and subsequent allergen delivery to the less–vascularized dermis, (2) increased convenience for patients due to the non-invasive (needle-free) and self-administrable application method, likely leading to improved compliance, (3) absence of additional potential irritant constituents (e.g., alum, preservatives), and (4) less cost–intensive than conventional AIT (81). Several clinical trials in AR patients used EPIT to deliver allergens of grass and birch pollen (82–85). The patch application time ranges from 8 to 48 h (86–89). EPIT might induce desensitization in patients with pollen sensitization, although at increased risk of local adverse events. However, more data are needed regarding patients with AR and indoor allergen sensitization.

### Local Nasal

Local nasal immunotherapy has been extensively investigated in the past 40 years and seems to be effective only on rhinitis symptoms. However, local nasal immunotherapy (LNIT) is not popular with patients due to local side effects that require topical nasal premedication for their prevention and difficulty of application (90). Currently, LNIT is not recommended for clinical use.

## Biomarkers

Allergen-specific immunotherapy is considered a precision medicine model for treating allergic diseases because of its individualized approach to treatment based on clinical and immunological profiles of each patient (91). Biomarkers are measurable indicators linking an underlying pathway to the phenotype or endotype of a disease. Identification of specific biomarkers that can identify responders, monitor treatment, predict the durability of therapeutic effects, and determine adverse event risk would aid clinical decisions and the delivery of targeted and effective treatments (91, 92). New potential biomarkers have been discovered with the emergence of advanced immunological, data-driven “-omic,” and molecular technologies (93, 94). Here, we briefly review a number of promising candidates that are being evaluated for AIT immune monitoring in the context of clinical trials as well as in real-world clinical settings (Table 1).

**Table 1:** The implementation of biomarkers in allergen-specific immunotherapy (AIT).

**Biomarkers**	**Assay**	**Advantages**	**Disadvantages**
IgE	Total IgE Specific IgE (serum/body fluid)	Serum sIgE is a gold standard of patient selection for AIT. Baseline sIgE/tIgE ratio may be a potential positive predictive marker for AIT.	The relationship sIgE/tIgE ratio and clinical outcome has been inconsistent.
IgG4	Specific IgG4 (serum/body fluid)	Elevation in serum sIgG4 is an indicator for compliance.	sIgG4 may not be related with clinical outcomes.
Inhibitory activity	IgE-FAB (serum/body fluid) ELIFAB	An association between serum inhibition activity and combined symptom-medication scores has been demonstrated.	Serum inhibition activity has restricted the availability requirement of specialized techniques.
Basophil activation	Basophil activation test via flow cytometry	*Ex vivo* test reflects the *in vivo* allergen-sensitized response.	The results are variable with inhibition being shown in some but not all studies. Standardized and optimized assays are needed.
Cytokines and chemokines	Serum/body fluid/*in vitro* cell culture-based by ELISA or Luminex	Serum and local cytokines and chemokines may be useful for exploring mechanisms of AIT and proof of concept at drug development.	Serum and local cytokines are at a low level in concentration.
Cellular markers	Immunophenotyping in *ex vivo* or *in vitro* activating cells, or tissue biopsy	Change in multiple cell subsets may be useful for exploring mechanisms of AIT.	There is not sufficient information to link the presence or function of cell subsets with clinical efficacy. The standardization for the identification of most cell types is deficient.
Clinical biomarkers	Allergen provocation test	Provocation tests have been used as surrogate markers to diagnose local allergic rhinitis and evaluate clinical response to AIT.	Allergen provocation cannot replace natural exposure in phase III clinical trials.

### Specific Immunoglobulins and Their Inhibitory Activity

Measurement of IgE is the first step in the diagnosis of atopic diseases. At present, detection of sIgE, either through measurement in serum or by an *in-vivo* skin prick test, and the manifestation of symptoms on exposure to the sensitizing allergen is the only criteria for allergy diagnosis and starting AIT (95). Several studies showed that the allergen-specific IgE (sIgE) and total IgE (tIgE) levels increase transiently during the initial stages of AIT but then return to their pre-treatment levels during the maintenance phase (96, 97). These trends seem to vary primarily with the duration of AIT and the time of sampling. A slow decrease in those levels may not be accompanied by a favorable clinical outcome (91). Several studies reported that the sIgE/tIgE ratio before AIT might predict the ultimate efficacy of AIT (91, 98, 99). A retrospective study in patients who underwent grass-pollen or HDM SCIT or SLIT found that the clinical response to AIT correlated significantly with the initial sIgE/tIgE ratio (*r* = 0.723, *p* < 0.0001). The sensitivity and specificity of the decision point for a serum sIgE/tIgE ratio of >16.2% were 97.2 and 88.1%, respectively (98). Others also found a similar correlation between the ratio and AIT outcome, but a small randomized controlled open-label study could not replicate those results (99).

Numerous studies have found that the IgG1 and IgG4 levels increase during AIT. Allergen-specific IgG4 (sIgG4) can compete with sIgE for allergen binding, thereby blocking allergen-IgE complex formation and preventing mast cell and basophil degranulation, IgE-dependent cytokine secretion from mast cells, binding of allergen to B-cell receptors on IgE^+^ memory B cells, and allergen presentation to T cells (100). A correlation between allergen sIgG4 and clinical outcomes has been reported in some but not all studies (91). Furthermore, sIgG4 levels do not always differentiate between responders and non-responders (101). Thus, an IgG4 increase during AIT may reflect compliance, not clinical efficacy. The absence of sIgG4 induction may also be indicative of poor compliance (91). The sIgG4/IgE ratio may monitor AIT progress and outcome, but it has shown inconsistent utility (102, 103). Intriguingly, sIgG4 fell back to its pre-treatment level within 1 year after discontinuation of AIT, but its inhibitory capacity for serum IgE persisted for several years, together with clinical benefits (104). That suggests that sIgG4 might have either higher avidity or higher affinity (105). Besides sIgG4, allergen-specific IgA (sIgA) is also induced during grass-pollen SLIT and HDM SLIT. sIgA and other subclasses of IgG may have a similar blocking function (97). There are only limited data regarding the roles of other IgG subsets, i.e., IgD and IgA, in serum.

IgE-facilitated allergen binding (IgE-FAB) is a highly reproducible flow cytometry-based bioassay that was developed to detect binding of allergen-IgE complexes to B cells that express surface low-affinity IgE receptor FcεRII (CD23). This bioassay is used to determine the antigen-presenting capacity of B cells to T cells (106). It has been developed as a surrogate for determining IgE-inhibitory activity during AIT (91). In addition to sIgG4, which is responsible for serum inhibition of IgE, there may be other factors that support serum inhibition of IgE because IgG4-depleted serum retained its blocking activity (107). These factors need further study. It was found that serum inhibitory activity determined by IgE-FAB showed potential to predict the clinical response (107). They were reported that changes from the baseline of IgE-FAB at the initiation of the maintenance phase and persist at least 1 year after AIT discontinuation associated with clinical manifestation (104). Inverse correlations were found between the symptom score, the rescue medication score, and the IgE-FAB result (35). These findings suggest that the serum inhibitory activity for IgE could predict the final efficacy of AIT as early as at the start of the maintenance phase of (105). To date, no data are available on the association between the initial level of serum inhibitory activity for IgE-FAB and responsiveness to AIT (91). An alternative test is the enzyme-linked immunosorbent-facilitated antigen binding (ELIFAB) assay, which follows the basic principles of a standard ELISA protocol and is able to detect the inhibitory activity for IgE after AIT (108). Although the IgE-FAB and ELIFAB techniques show good clinical efficacy correlation for AIT, they are both complicated, and their use is limited to specialized laboratories (91, 92).

In addition, the sIgG subclass and sIgA levels can be detected in the nasal lavage of allergic patients (109). An increase in the IgG4 level was significantly associated with reduced nasal sensitivity. A study of grass-pollen AIT patients demonstrated that the nasal sIgG4 level increased during the pollen season. The inhibitory activity for IgE-FAB of the nasal fluid and serum were significantly increased in the SCIT group and correlated with the total symptom improvement, indicating that sIgG4 produced locally in the nasal mucosa can be a potential biomarker for AIT efficacy (35, 110). Moreover, a recent study compared the nasal and systemic grass-pollen sIgG4, sIgA1, and sIgA2 responses during 2 years of SCIT and SLIT and 1 year after treatment discontinuation. Production of sIgA was shown to be a major biological difference between SLIT and SCIT. Although SCIT induced higher specific sIgG4 levels than SLIT, SLIT led to higher sIgA levels both in serum and nasal fluid. The level of sIgA1 in nasal fluid correlated with the suppression of nasal symptoms of SLIT during nasal allergen challenge. sIgA production may therefore represent a distinct mechanism by which SLIT achieves its therapeutic effects (32).

As stated in the European Academy of Allergy and Clinical Immunology (EAACI) Position Paper, serum-based biomarkers are beneficial for selecting patients for AIT. An elevated sIgE/tIgE ratio is a potential positive predictive marker for AIT. The sIgG4 level is proposed to be an indicator of compliance of patients, but it shows no association with the efficacy of AIT. Serum inhibitory activity for IgE, determined by IgE-FAB rather than the level of sIgG4, might be associated with the symptom and rescue medication scores and predictive of the clinical outcome (91).

### Basophil Activation

To determine allergen sensitization, basophils are incubated with a specific allergen, followed by an examination for degranulation. Activation of basophils leads to upregulation of surface markers, which is indicative of sIgE functional activity. Several surface markers indicate basophil responsiveness and histamine release, i.e., CD63, CD203c, CD13, CD107a, and CD164. Intracellular histamine-binding fluorochrome-labeled diamine oxidase can be quantified by flow cytometry. Blocking antibodies, such as sIgG4, are augmented during AIT. They inhibit cross-linking of allergens to sIgE bound to the surface of basophils and hereby suppress basophil activation (111).

The findings regarding basophil activation during AIT in placebo-controlled trials are inconsistent (91). Some studies describe reduced basophil activation after AIT with the decline correlating with clinical score improvement (112, 113), while others failed to show suppression (114). One study found no significant changes in basophil activation after SLIT, despite induction of sIgG4 (114). These contrasting findings may be explained by differences in the immunotherapy route, with SLIT possibly being less effective than SCIT in inhibiting basophils. Also, the methods used to measure the markers of basophil activation may alter the outcome (91).

### Cytokines and Chemokines

The mechanism of induction of immunological tolerance by AIT is the redirection of the Th2 phenotype toward a Th1 and Treg phenotype. One would anticipate decreases in Th2 cytokines (e.g., IL-4, IL-9 13, IL-19) and chemokines (e.g., eotaxin), and upregulation of Th1 (e.g., IFN-γ) and regulatory cytokines (e.g., TGFβ, IL-10) (100, 105). However, serum cytokine measurement is difficult due to their low levels, which are often below the limit of detection of current methods. Furthermore, relationships between serum cytokines and the clinical outcome of AIT have not been elucidated (91). Shifts in cytokine production by CD4^+^ T cells following AIT are quantifiable through *in vitro* stimulation of PBMCs from patients by treating them with allergen extracts at both the protein and the transcript levels (92). High levels of IL-10 transcripts in T cells of patients with HDM allergy predicted the success of AIT (115).

Local rather than serum levels of cytokines may be predictive of the clinical efficacy of AIT. Local cytokine production following nasal allergen challenge may be an important treatment-related indicator (91). A cross-sectional study found lower concentrations of Th2 cytokines and chemokines in the nasal fluid after nasal allergen challenge following successful AIT compared to untreated controls (110). In a double-blinded randomized controlled trial, both SCIT and SLIT led to a decrease in Th2 cytokines, including IL-4, IL-5, and IL-13 in the nasal fluid after nasal allergen provocation after 2 years of continuous AIT, and those changes were associated with improvement in the clinical symptoms (19). At this stage, local and systemic cytokines, and chemokines are not practical as biomarkers in clinical practice. However, nasal cytokines can serve as markers of the immunological response and be used for proof of concept in drug development (91).

### Cellular Markers

Tregs play a key role in immune tolerance to an allergen after AIT (100, 105). There are two main types of Tregs, i.e., natural regulatory T cells (nTregs) that express FOXP3^+^ CD4^+^ CD25^+^, and inducible Treg cells (iTregs) generated in the periphery under different tolerogenic conditions that produce regulatory cytokines such as IL-10 and TGF-β. Different studies have shown the immunomodulating properties of both allergen-specific nTregs and iTregs in blood and tissues after SCIT and SLIT, suggesting that there is a commonality between these subgroups of Tregs (91). In AIT, initiation of peripheral T-cell tolerance presents anti-inflammatory cytokines IL-10 and TGF-β. An increased number of IL-10-expressing T cells during pollen season and a seasonal increase in TGFβ^+^ T cells correlated, respectively, with an increase in the serum IgG4 level and an increase in the peripheral circulating IgA concentration (116). Upregulation of activated allergen-specific Tregs (Der p1-specific FOXP3^+^ Helios^+^IL-10^+^ Tregs) and downregulation of a dysfunctional allergen-specific Treg cell subset (ILT3^+^ Tregs), associated with improved clinical response, were recently described in a study of HDM-SCIT treated patients (29). Identification of cell subsets, proteins, transcripts, and epigenetic biomarkers may suggest the prognosis. A recent randomized controlled study investigated epigenetic modification in the FOXP3 promoter region and found that methylated CpG sites within the FOXP3 locus of enriched peripheral memory Treg cells were reduced after SLIT treatment, leading to immune tolerance (117).

A novel effector subgroup of Tregs, i.e., Tfr cells, was recently identified. Tfr cells can suppress Tfh cell-mediated B-cell activation and antibody production (17). Recent evidence shows that AIT modulates the balance between circulating Tfh and Tfr, with Tfr as a potential biomarker for AIT efficacy (25, 27). A study showed increased numbers of circulating Tfr cells, with improved suppressive function, in AR patients after HDM SCIT (23). Therefore, a better understanding of Tfr cells will help in the development of novel strategies for AIT.

The involvement of B cells in allergen tolerance is mainly through regulatory B cells (Bregs). Bregs are a subset of B cells that have immunosuppressive and anti-inflammatory properties, predominantly via the release of IL-10, auxiliary Treg differentiation, IgG4 production, and inhibition of the inflammatory responses facilitated by T cells and DCs (118). IL-10-producing Bregs have been isolated from bee venom-tolerant subjects, and they suppress the proliferation of bee venom-specific T cells. Bee-venom immunotherapy (VIT) increases the number of phospholipase A2 (PLA)-specific IL-10-producing Bregs to a level comparable to in healthy beekeepers. Interestingly, both groups have high levels of PLA-specific IgG4-switched memory B cells, plasmablasts, and PLA-specific CCR5-expressing B cells (7). Recently, a study of grass-pollen SCIT showed an increase in the number of IL-10^+^ Bregs that was associated with an increase in the sIgG4 level in the nasal fluid (35). Furthermore, successful HDM SCIT-treated patients were shown to have heightened frequencies of IgA and IgG4-expressing allergen-specific B cells, plasmablasts, and IL-10^+^ and/or IL-1RA^+^ Bregs (29).

Growing evidence has proven the role of innate immunity in allergic diseases, and there is a heightened focus on how AIT alters ILC2s to induce tolerance. Peripheral ILC2s were suppressed by grass-pollen SCIT. The level of ILC2s correlated with the severity of self-reported symptoms during the pollen season (39). Likewise, ILC2s in the peripheral blood of SCIT-treated, HDM-allergic AR patients were reduced compared with the untreated group (42). More recently, a subset of ILC2s able to produce the regulatory cytokine IL-10 was described (43, 45), and they attenuated Th responses and maintained epithelial cell integrity. IL-10^+^KLRG1^+^ ILC2s were fewer in patients with grass-pollen allergy compared to healthy subjects. The ability of ILC2s to produce IL-10 was restored in patients who underwent grass-pollen SLIT. Moreover, symptom severity correlated inversely with the number of IL-10-producing ILC2s after immunotherapy (46).

Dendritic cells are specialized antigen-presenting cells with the ability to integrate a variety of incoming signals and subsequently orchestrate adaptive immune responses. Molecular markers associated with polarized monocyte derived DCs that support the differentiation of either effector Th1, Th2, Th17, or regulatory CD4^+^ T cells (termed DC1s, DC2s, DC17s, and DCregs, respectively) have been identified by comparative transcriptomic and proteomic analyses (91). AIT modulates DCs by up-regulation of DCreg markers and down-regulation of DC2 markers (54). There was also a significant increase in DCs with the DCreg phenotype [assessed by mRNA expression of stabilin-1 and complement component 1Q (C1Q)], with enhanced capacity to generate IL-10 with diminished IL-12 in peripheral blood samples from responders to SLIT (54).

At this stage, no cellular biomarker can serve as a biomarker for monitoring AIT in clinical practice. However, biomarkers may be valuable as indicators of immunological responses in drug development and in AIT mechanistic studies (91).

### Clinical Biomarkers

Allergen provocation tests, such as conjunctival provocation tests, nasal provocation tests, and environmental exposure chambers, are used to evaluate target organ responses. APTs are commonly used in clinical practice to assess allergen-specific reactivity of patients and the clinical relevance of IgE-mediated sensitization (91, 119). They are a vital tool for the diagnosis of local AR (92, 119, 120). These tests can also be used as *in vivo* methods for stratifying patients when investigating the therapeutic effects in AIT trials. Allergen provocation tests (APTs) permit better standardization of procedures, control of environmental factors (temperature, humidity), and avoidance of variability caused by seasonal variations in pollen exposure. They are used as surrogate markers of the clinical response to AIT. APTs are recommended to provide insight into the mechanisms of AIT and biomarkers at both the local and systemic levels (91, 119). The European Medicines Agency (EMA) suggests APTs as primary endpoints in proof of concept and dose-finding trials of AIT (phase II) before proceeding to phase III AIT trials. However, APTs cannot be substituted for assessing symptoms and requirements for rescue medication during natural allergen exposure in phase III trials (91).

## The Efficacy in AR

Allergen-specific immunotherapy has been shown to be useful for the long-term reduction of medical expenses because of its sustained, disease-modifying effects. After administration for 3 to 4 years, both SCIT and SLIT effectively improved allergic rhinoconjunctivitis (121), and asthma (122). The rate of new antigen sensitization after 2 years was significantly lower in patients undergoing AIT than in non-AIT patients. Moreover, there was a 2–3-fold reduction in the risk of development of asthma for 2–7 years after stopping AIT (123). Some studies also found that there might be a lower prevalence of allergy in children born to mothers who underwent AIT during pregnancy. A total of 56 homogeneous studies between 2003 and 2013, including SCIT and SLIT, concluded that the recovery rate in AIT groups was 53.67-fold higher than in the placebo groups (124). The rate of reduction of symptoms and the medication score was as high as 80% in SCIT for seasonal AR in many randomized, placebo-controlled trials (RPCTs). Accordingly, the efficacy of AIT depends on the allergen dose and treatment duration. The clinical results have shown a high degree of heterogenicity and responsiveness in individuals. The immunological response was related to the personal dose (125), and long-term improvement after discontinuation was related to the treatment duration (126). There are no definitive diagnostic tools or markers for identifying responder patients, so current practice suggests that physicians discontinue AIT if there is no clinical response after 18–24 months (127). However, a standardized extract dose and clinical data are not available for all extracts. The extracts in each country have different potency, allergen dose, allergen mixtures, and adjuvants. Moreover, data for direct comparisons of AIT and pharmacotherapy are lacking because of a dearth of head-to-head studies.

### Comparison of SCIT and SLIT

Subcutaneous immunotherapy and SLIT differ in schedules, route, frequency, amount of allergen, up-dosing, and maintenance dosage. Nevertheless, clinical efficacy is evaluated in the same way by using subjective and objective parameters. SCIT has demonstrated benefits in children and adults with AR. Symptom reduction has persisted for many years after stopping treatment. Meta-analysis comparing SCIT and SLIT revealed both to be effective for seasonal AR. In perennial disease with HDM allergy, SCIT also showed benefit, but SLIT was doubtful (128). Analysis of all randomized studies of SCIT generated a dose-response curve (129). Effective doses were associated with the amount of allergen, but side effects of SCIT also increased.

Meanwhile, SLIT showed a wide range of effective doses (130). Some studies showed improvement in the second year of treatment (131). A meta-analysis that compared the efficacy of SCIT, SLIT tablets, and SLIT drops for grass AR found no difference between SCIT and SLIT tablets, whereas SLIT drops were less effective than the tablets (132). The early study in 20 adults mono-sensitized to grass and treated with either SLIT drops or SCIT showed that the combined symptom and medication scores decreased by at least 50% in both groups compared with the placebo, whereas sIgG4 changed only in the SCIT group (133). However, direct comparisons of 11 randomized studies showed that SCIT was more effective than SLIT compared with the placebo (134). A direct pairwise meta-analysis of 26 double-blind randomized controlled trials to compare the efficacy of HDM AIT using SLIT drops, SLIT tablets, and SCIT in patients with perennial AR to HDM showed that the symptom score was more significantly decreased with SCIT than with SLIT drops or SLIT tablets (135).

However, more head-to-head, large, and well-designed studies are needed to compare the effectiveness of SCIT and SLIT. A head-to-head comparison of SCIT and SLIT in the grass-pollen allergic mice showed that SCIT suppressed Th2 inflammation and induced neutralizing antibodies, whereas SLIT suppressed allergen-induced airway hyper-responsiveness and induced a grass-pollen-specific IgG2a response (136). An evaluation of AR patients who changed from SCIT to SLIT for a variety of reasons found similarity of symptoms in 75%, while SCIT was preferred by 8% and SLIT by 17% (137). Indirect comparisons tend to conclude the superiority of SCIT due to the rapid improvement and immunological change. Adverse reactions to SCIT included a higher risk of local and systemic allergic reactions compared with SLIT. Therefore, the risk with SCIT correlated with a larger injection volume, multiple allergens per shot, and a higher extract concentration. Retrospective data showed that 23% of SCIT patients experienced systemic reactions after injection (138). On the other hand, the safety of SLIT was better. Reactions were recorded in 10–15% of SLIT patients, and most were mild reactions in the early phase of treatment.

### ILIT and EPIT

Several clinical trials testing the efficacy of ILIT in the treatment of grass, birch, and cedar-pollen, and cat–dander allergies have shown high therapeutic efficacy (75, 139–141). A systematic review and meta-analysis of 11 randomized controlled trials and 2 cohorts showed short-term benefits of ILIT for seasonal allergic rhinoconjunctivitis (142). ILIT improved the composite score and visual analog scale and increased sIgG4 levels but did not change the quality of life or sIgE levels. A recent study found that 3 injections without an annual booster achieved a substantial reduction in allergic symptoms and use of rescue medication during a 3-year follow-up (143).

Epicutaneous immunotherapy seems effective and safe for rhino-conjunctivitis. In adults with timothy-grass-pollen allergy, applying a Phl p 5 patch for 6 weeks reduced the allergic symptoms and medication use in the treatment group compared to the placebo group (89). EPIT efficacy was dose-dependent, but a high dose was associated with local skin inflammation (144). EPIT for HDM AR was studied in an animal model (145). A number of questions remain, such as the standard dose, time of treatment, type of antigen, and placebo effect, and are in need of further study.

### Comparison of AIT for Seasonal and Perennial AR

The meta-analysis in AIT studies found SCIT to be significantly effective in patients with seasonal (93) and perennial AR who were sensitive to HDM (146). SLIT tablets for timothy grass, a 5-grass mix, ragweed, and HDM showed efficacy in relieving symptoms in America and Europe. Asthmatic child patients who underwent SCIT treatment for more than 3 years showed control of the symptoms of seasonal AR for 7 years after discontinuing the AIT (123). In patients unresponsive to regular drug therapy, SCIT reduced symptoms and medication in pre and co-seasonal immunotherapy (147, 148). Subjects started SCIT at least 8 weeks before the season and continued for at least 16 weeks (149). Allergy drops for birch, alder, and hazel also showed benefits in Europe. In pooled analyses, Durham et al. (150) showed that, compared with the placebo, nasal symptoms improved in seasonal AR by 4–27.2% with the 6–timothy–grass SLIT tablet (overall improvement 16.3%) and by 15.2–18.8% with the 2–ragweed SLIT tablet (overall improvement 17.1%).

For perennial AR, nasal symptoms improved by 16.1% with HDM SLIT tablets relative to the placebo. The combined symptom and medication score (CSMS) also decreased significantly in patients using the HDM SLIT tablets for 1 year (99). Thus, HDM SLIT tablets were more beneficial than all pharmacotherapy regimens in perennial AR trials. Medication reduction with SLIT tablets for perennial AR was 1.5–2-fold compared to seasonal AR. SCIT for cat and dog allergies yielded no meaningful data because of low potency and variable standardization of allergens.

### Comparison of Mono-Allergen and Poly-Allergen AIT

There is no standardized approach to AIT for poly-sensitized patients. In Europe and Asia, mono-allergens have predominantly been used by choosing allergens that correspond with the symptoms. However, in the United States, all relevant allergens have been given to allergic patients (separate shots or mixed shots). Poly-allergen AIT is performed by administering mixed extracts at a single body site or single extracts at different body sites, simultaneously or at different times. Poly-allergen extracts were effective in SCIT (151), and the therapy is safe if administered in an appropriate setting. However, data for poly-allergen SLIT are scant. Therefore, there has been no head to head clinical outcome comparisons of mono-allergen and poly-allergen SCIT or SLIT. A meta-analysis study of HDM AIT in mono and poly-sensitized patients with AR found no significant differences in the nasal symptom score, medication score, or quality of life between the groups. The study concluded that single-allergen AIT using HDM was clinically effective for both mono and poly-sensitized AR patients (152). Component-resolved diagnosis is essential for avoiding the inclusion of irrelevant allergens in mixed shots. An Expert Committee recommended limiting mixtures to 2 or 3 extracts for patient safety (153). The European AIT guidelines recommend that poly-sensitized patients who are poly-allergic to taxonomically–related homologous allergens be administered either a single allergen or a mixture of homologous allergens (95). Moreover, patients who are poly-allergic to non-homologous allergens should be started on AIT with either the allergen responsible for most of their AR symptoms or separate treatment with the two clinically most important allergens (154).

### The Efficacy in AR With Co-morbid Disease

#### Asthma

Allergen-specific immunotherapy has shown effectiveness against allergic asthma. The common allergens in patients with allergic asthma are similar to AR, including HDM, grass pollen, tree pollen, and animal dander (122). A recent systematic review and meta-analysis of 98 studies showed that SCIT and SLIT significantly reduced short-term symptom scores and medication use in patients with allergic asthma (155). SCIT improved the quality of life (QoL) (156–158), whereas SLIT showed variable results in patients with allergic asthma (155). SCIT did not reduce asthma exacerbation, defined as the number of oral corticosteroids needed to restore asthma control (159), but SLIT also showed inconclusive results. A large randomized controlled trial of HDM SLIT tablets in patients with allergic asthma found the treatment extended the time to exacerbation during inhaled corticosteroid (ICS) reduction in suboptimally–controlled asthma (160). Importantly, there were no reports of severe systemic allergic reactions in SLIT patients (160). Nevertheless, data are limited regarding the ability of SLIT to suppress asthma exacerbation (160, 161).

Allergen-specific immunotherapy significantly improved the forced expiratory flow at 25–75% but not the peak expiratory flow rate (PEFR) or forced expiratory volume in 1 s (FEV1) (155). SCIT improved bronchial hyper-reactivity, but nothing was reported regarding SLIT (155). Conversely, SCIT caused more systemic adverse effects than SLIT (122, 155). Some cohorts showed a benefit of AIT in preventing the onset of asthma in allergic rhinitis patients (162, 163). The EAACI guideline recommends AIT as an add-on to regular asthma therapy in adults with controlled or partially-controlled HDM-driven allergic asthma (164). “Controlled asthma” is defined as daytime symptoms <2 times/week, no night awakenings, relief is needed for symptoms <2 times/week, and no activity limitation due to asthma. “Partially-controlled asthma” is defined as failure to meet the first 2 criteria above. The updated asthma guideline recommends SLIT in adult HDM AR patients with asthma that is suboptimally–controlled despite low to high dose inhaled corticosteroid and FEV1 >70% (160, 165).

#### Atopic Dermatitis

Many studies have been conducted in AR patients with or without asthma who also have atopic dermatitis (AD). Some patients showed improvement in AD symptoms, and no patients became worse (166, 167). A systematic review and meta-analysis reported a moderate level of evidence for effectiveness in improving the total SCORing Atopic Dermatitis (SCORAD) index over 18 months of SCIT (168). No fatal or near-fatal adverse events were reported in any of the studies assessed. SLIT also improved the total SCORAD (169, 170). However, another systematic review of 12 eligible trials (6 SCIT and 4 SLIT) found no significant differences in the disease severity score or eczema symptoms (171). Therefore, large controlled and randomized clinical trials are needed to study this more. Nevertheless, AIT may be an effective treatment option for selected AD patients (172).

#### Sinusitis and Nasal Polyps

Allergen-specific immunotherapy also shows good efficacy in AR with sinusitis. A survey study in the United States showed a 72% decrease in days lost from work, a 26% reduction in the use of medications per year, and a mean reduction of 51% in the overall symptom score in sinusitis patients who underwent AIT (173). In addition, AIT for allergic fungal sinusitis resulted in significant improvement in the endoscopic disease score and chronic sinusitis survey symptom score and decreased systemic corticosteroid use (174).

## Duration of AIT

Many randomized controlled trials show long-term efficacy in improving clinical and immunological change after SCIT and SLIT. Continuous SCIT for 3–4 years resulted in 3 years of persistent improvement in the clinical condition and medication (147, 175, 176). In the SLIT study, 3 years of grass-pollen sublingual drops showed benefit for only 1 year after stopping treatment (177). Three years of grass-pollen SLIT tablets showed a 20–30% reduction in symptoms and rescue medication for 2 years after discontinuation (147, 178–181). When AIT was administered for less than 3 years, allergic symptoms usually relapsed 1 year after discontinuation.

Patients undergoing AIT for more than 3 years showed clinical efficacy beginning after 1 year of treatment (19, 176, 177, 180, 182). A comprehensive 5-year prospective controlled trial that compared 3- and 5-year HDM SCIT found significant decreases in the rhinitis severity score, asthma severity score, and visual analog scale in both groups after 3 years. Moreover, the AIT benefit was maintained in both groups at 5 years (183). All the above evidence suggests that the duration of both SCIT and SLIT should be at least 3 years for long-term clinical benefit.

## Adjunctive Therapies in AIT

Adjunctive therapy in AIT refers to the use of another treatment together with AIT. Its purpose is to improve the efficacy of AIT and decrease its adverse effects (Table 2).

**Table 2:** AIT and adjunctive therapy.

**Adjunctive therapy**	**Immunological mechanism**	**Clinical benefit**	**References**
Vitamin D (VitaminD2 and D3)	1. Decrease DCs function by stimulating IL-10 production. 2. Increase production of Treg cells 3. Regulate innate and adaptive immune responses.	1. Improve symptoms in the patients with AR and allergic asthma patients. 2. Laboratory improvement of regulatory cells and decrease type 2 inflammatory cells	(23, 184–188)
Anti-IgE	Restore pDCs to Treg cells	1. Decrease allergic symptoms, rescue medication during seasonal exposure. 2. Decrease adverse events from immunotherapy, especially in high-risk asthma and rush protocol.	(189–194)
Anti-IL5 and Anti-IL-5 receptor	No current study using Anti-IL5 receptor or anti-IL5 monoclonal antibody as adjuvant therapy to AIT in human		–
Anti-IL4/IL-13 receptor	Monoclonal antibody against IL-4 receptor	Do not improve clinical response compared to AIT alone	(195)
Probiotic	1. Increase Treg cells, IgA antibodies production, and activity of DCs. 2. Conversion Th2 to Th1 response.	Additional AIT treatment with strain-specific probiotics might help clinical improvement in allergic patients.	(196–199)

### Vitamin D

Vitamin D is a major substance that enhances human immunity (200). Vitamin D2 is converted into active vitamin D3, which regulates innate and adaptive immune responses (184). Active vitamin D3 enhances IL-10 production from DCs and induces Tregs (185). The clinical efficacy of AIT was increased when vitamin D was sufficient (201, 202). Skin test reactivity to grass pollen was significantly reduced by grass-pollen AIT with adjunctive vitamin D supplementation compared to the placebo (203). Moreover, children with grass-pollen AR who underwent AIT with vitamin D showed a reduced symptom-medication score and improved lung function compared to the placebo (204). However, the role of vitamin D in AR remains controversial (205).

### Monoclonal Antibodies

Omalizumab is a monoclonal antibody that binds to the Fc portion of the IgE molecule. In *in vitro* testing, omalizumab also restored pDCs to Tregs (189). Combined SCIT with omalizumab reduced symptoms and rescue medication during seasonal allergen exposure compared to SCIT alone (190). Moreover, omalizumab reduced the adverse effects of AIT, especially in high-risk asthma patients and with the rush AIT protocol (191–193). Dupilumab is a monoclonal antibody against the IL-4 receptor. A recent study found that AIT combined with dupilumab did not improve the clinical response compared to AIT alone (195). There have been no studies using anti-IL5 receptor or anti-IL5 monoclonal antibodies as adjunctive therapy to AIT in humans.

### Probiotics

Probiotics have been proven beneficial for the immunological system. Some species have been shown to increase Tregs, IgA antibody production, and the activity of DCs. Thus, probiotics can help reduce the risk of immunologically-mediated disease, including Th2-mediated allergic responses that play a significant role in allergic diseases. *Lactobacillus* and *Bifidobacterium* are the main genuses used for the preparation of the products tested in several studies. Strain-specific probiotics were used for adjunctive treatment of AIT, specifically either probiotics or recombinant probiotics. However, the data are still limited. Probiotics may be ineffective after enzymatic degradation of allergens by the oral route. Combination recombinant probiotics producing the allergoid may be better to use only an allergoid for AIT treatment of AR patients because of a safer and more effective. Most studies of recombination probiotics in murine models as pre-clinical studies showed reduced sensitization in both newborn and adult mice (196). Intranasal vaccination of adult mice with *Lactococcus lactis* strains resulted in decreased sIgE antibodies and increased sIgA antibodies (197). Also, oral treatment of adult mice with *Lactobacillus acidophilus* strains increased sIgG antibodies (197). It appears that recombinant probiotics can modulate the immune response, shifting it toward a Th1 and Treg-specific immune response, but it remains unclear whether long-lasting immunological tolerance is induced.

Several human studies have shown that probiotics reduce symptoms and improve the quality of life in AR patients. A systematic review by Zajac et al. (206) found that the duration of probiotic administration varied from 4 weeks to 12 months. However, probiotics did not affect either tIgE or sIgE. In another study, SLIT with adjunctive probiotic treatment showed significantly higher Tregs than in the SLIT only group (198). Overall, the mechanism and efficacy of probiotics in AR management remain unclear. Nevertheless, probiotics have the potential as adjunctive therapy in AR management.

## Adjuvants in AIT

Adjuvants are substances that precipitate with an allergen extract in AIT vaccines (Table 3). The aim is to skew a robust Th2 immune bias toward the cytosolic inflammatory pathway for enhanced antigen cross-presentation and IgG production or toward the vacuolar pathway with a clear Th1 shift and active tolerance (226). Also, adjuvants can prevent the too rapid systemic distribution of allergens at the injection site.

**Table 3:** Adjuvant in AIT.

**Vaccine adjuvant therapy**	**Immunological mechanism**	**Clinical benefit**	**References**
TLR agonist (MPL)	1. Reverse immune toward Th1/Treg response. 2. Significant decrease IgE level and increase production of IgG4 level.	Improve symptom score.	(207–209)
TLR agonist (LPS)	1. Promoted human DCs to produce IL-12p70 and IP-10 and potent Th1-biased stimulus. 2. Downregulate Th2 responses by reducing IL-13.	Clinical benefit in human needs to study more.	(207, 210, 211)
CPG-ODNs	1. Shift human allergen-specific Th2 cells to Th1/Th0 phenotype. 2. Reduce Th2 inflammation and IgE secretion. 3. Increase in regulatory Treg cells.	1. Reduce symptoms of allergic asthma in the mouse model. 2. Show long-term clinical efficiency in patients with ragweed AR in phase II clinical trial but in phase III controlled clinical trial show lack of success in efficiency.	(212–215)
Aluminum hydroxide	1. Increased allergen immunogenicity and IgG and IgE titers 2. Recruitment and activation of APCs at the injection site.	Inconclusive	(216–219)
Calcium phosphate	Adsorb antigens and increases IgG levels.	Induce local adverse reactions.	(220, 221)
Microcrystalline tyrosine	Increased IgG production and limited increases of IgE levels	Safe in using as adjuvant of AIT in humans.	(222)
Fungal compounds	Stimulate the innate immune system and induce cytokine for the adaptive immune system.	Inconclusive	(223)
Heat-labile toxin (LT) from *Escherichia coli*	Stimulate the innate immune system	Inconclusive	(224)
Parasite molecules	Suppression of host antigen-specific immune response	Inconclusive	(225)

### Toll-Like Receptor Agonists (TLRs)

Toll-like receptor ligands comprise the innate immune system that responds to pathogen-associated molecular patterns (PAMPs). AIT with adjuvant TLR shows benefits and can reverse allergic inflammation (207). TLR4 and TLR9 have been tested for TLR-activating properties in allergic diseases (208, 227). TLR4 ligands are monophosphoryl lipid A (MPL) and lipopolysaccharide (LPS) (208). MPL can promote a shift in the immune response toward a Th1/Treg response (208). MPL is now being investigated in a clinical phase III study by both subcutaneous and sublingual routes (209). Laboratory markers showed a significant decrease in the IgE level and increased production of IgG4. The symptom score also improved more than with AIT without MPL (209). TLR4 has been used as an adjuvant in vaccines for cancer and infection. However, TLR4 as an adjuvant of AIT for AR or asthma is unclear, but LPS has been used to stimulate TLR4 in many animal studies. LPS can promote human DCs to produce IL-12p70 and IP-10 and is a potent Th1-biased stimulus (210). *In vitro* models using human cord blood cells also showed downregulation of Th2 responses due to reduced IL-13 after LPS administration (211). Clinical studies are needed to determine the effectiveness of LPS as an adjuvant of AIT in humans.

### CPG-ODNs

Unmethylated deoxycytidyl-deoxyguanosine oligodeoxynucleotides (CpG-ODNs) are PAMPs that mimic bacterial DNA. CpG-ODNs stimulated TLR9 (228). CpG-ODNs were previously considered to be potential vaccine adjuvants (227). CpG-ODNs can shift human allergen-specific Th2 cells to a Th1/Th0 phenotype. In a murine model, CpG-ODNs decreased Th2 inflammation and IgE secretion (212) and increased Tregs (213). The USFDA approved CpG-ODN as an immunoadjuvant in the hepatitis B vaccine (229). CpG-ODNs are also used as immune modulators in many cancer immunotherapies. Recently, AIT cat allergen Fel d 1 with high-dose CpG-ODNs reduced all allergic symptoms in a murine model. Moreover, pDCs were increased and migrated from the injection site to periphery sites (213). CpG-ODNs showed long-term clinical effectiveness in patients with ragweed AR in phase II clinical trials (214), but its efficacy was lacking in phase III controlled clinical trials (215). A randomized controlled trial in humans is needed to generate more information.

### Aluminum Hydroxide

Aluminum hydroxide is the most common adjuvant used in vaccines and AIT (230). Aluminum hydroxide in AIT can induce allergen immunogenicity and increase IgG and IgE titers (216), and create a sustained-release antigen depot leading to greater safety (230). Aluminum hydroxide also induced greater inflammation due to the recruitment and activation of APCs at the injection site (217). The adverse effects of aluminum hydroxide constitute a significant problem: acute and chronic inflammation at the injection site was found in more than 15% of AIT patients (218). At present, there is no clear consensus regarding the benefit and serious adverse events of using aluminum hydroxide as an adjuvant in AIT (219).

### Calcium Phosphate

Calcium phosphate is a mineral salt that could be used as an adjuvant in AIT (216) because it can adsorb antigens and increases IgG levels (220). However, it might cause local adverse reactions. Calcium phosphate will be considered as an alternative to aluminum hydroxide, but with lower adjuvant activity (221).

### Microcrystalline Tyrosine

Microcrystalline tyrosine has been used as an immunomodulator and adjuvant. The product released from the injection site is L-tyrosine. Microcrystalline tyrosine (MCT) increased IgG production while suppressing the IgE level (222). L-tyrosine is safe when used as an adjuvant of AIT in humans. However, caution is required in regard to possible tyrosine metabolism disorder (222).

### Fungal Materials

Compounds of fungal origin, i.e., fungal immunomodulatory proteins (FIP), such as glycophosphopeptical, have been shown to stimulate the innate immune system via non-specific receptor recognition molecules and induce cytokines for the adaptive immune system (223). However, the results for FIP are inconclusive because most studies of FIP add-on to AIT have shown no superior clinical improvement in AR patients compared to AIT alone.

### Heat-Labile Toxin

Patch delivery of a combination of birch-pollen allergen and rBet v 1 with the heat-labile toxin from *Escherichia coli* was superior in inducing allergen-specific IgG compared with subcutaneous alum-adsorbed rBet v 1 in an animal model (224).

### Parasite Proteins

Helminths can evade host immunity by suppressing the antigen-specific immune response of the host. For example, *Brugia malayi* TGF-β homolog-1 and *Brugia malayi* TGF-β homolog-2 can bind to human TGF-β receptor (225) and mimic human TGF-β. Such parasite molecules might be able to serve as adjuvant carriers for AIT in the future.

## Different Types of AIT

It is hoped that advanced technologies will be able to be combined with AIT to achieve greater efficacy and safety. The aims are IgE-activity reduction, allergenicity reduction, and induction of allergen-specific blocking IgG antibodies. Methods would include bypassing IgE, targeting T cells, modification of natural extracts, and use of multiple recombinant allergens.

### Component-Resolved AIT

The proportion of poly-sensitized AR patients has increased along with cross-reacting allergens (i.e., profilin, polcalcin, lipid-transporting proteins, tropomyosin, etc.). Allergen sensitization varies among different age groups, study populations, and geographical regions. In many countries, allergen extracts for immunotherapy still use whole extracts. Component-resolved diagnostics (CRD) has been brought to identify sensitization to allergenic proteins and to improve AIT efficacy in poly-sensitized patients (231). In a murine model of cockroach allergy, component-resolved immunotherapy using Per a 9 found reduced levels of Per a 9 sIgE, whereas sIgG1 and sIgG2 antibodies did not show significant change (232). In a human study, 1,263 Spanish patients with seasonal AR to grass and olive pollens underwent AIT based on skin prick tests in 73% or CRD IgE antibodies in 56.8% of the patients. The results showed that AIT prescribed based on CRD was more accurate and reduced the cost of immunotherapy (233).

### Recombinant Proteins

Recombinant allergen-based vaccines that use allergen-encoding DNA have been developed for both SCIT and SLIT. The aim is less induction of IgE response and good induction blocking allergen-specific IgG antibodies. Advances in molecular cell biology enable the use of recombinant wildtype allergens (which contain mainly conformational IgE epitopes that eliminate the problem of poor quality of natural allergens), recombinant hypoallergens (which, by DNA technology, convert allergens to abolish IgE activity but leave the T–cell response), and recombinant fusion proteins (carrier proteins and non-allergenic allergen-derived peptides that contain tolerogenic epitopes) (234). Significant benefits accruing from recombinant proteins are more effective immune responses and fewer systemic reactions following AIT. The recombinant hypoallergen Bet v 1 was reported to significantly increase Bet v 1-specific IgG1 and IgG4 antibody levels and decrease the medical symptom score in AR patients compared with non-AIT groups (235). Long-term efficacy was seen in patients with allergic rhinoconjunctivitis more than 3 years after completion of treatment (236). Non-allergenic peptides from the major grass-pollen allergen and the major HDM allergen induced allergen-specific IgG antibodies in allergic patients. A novel recombinant fusion protein might be able to be used with inactivated *Escherichia coli* as the expression system, and rhinovirus-derived coat protein or hepatitis B as a carrier protein. However, results in humans are inconclusive due to scant data, and variations in extract preparation, dosing, the dosing interval, and the reaction products.

Recent technology has already been developed for AIT with allergen hybrids or mosaic antigens by fusion of different protein sources, such as pollen, animal dander, and various foods. The hybrid allergens are modified to be hypoallergenic while still being able to induce T–cell tolerance. A Fagales pollen hybrid (birch, hazel, alder, oak, and hornbeam) molecule for AIT was more efficient in raising a T-cell response and showed lower IgE-binding capacity compared with the crude extracts in a murine model (237). In a study in rabbits, a recombinant hybrid molecule consisting of the major birch allergen (Bet v 1) and grass-pollen allergen (Phl p 5) increased IgG antibodies and reduced allergenicity (238). A clinical trial that administered a vaccine containing major grass-pollen allergens (Phl p 1, Phl p 2, Phl p 5, and Phl p 6) to patients with allergic rhinoconjunctivitis found significantly increased grass-pollen-specific IgG and a decrease in the total nasal symptom score (TNSS) (239). However, a small study in patients with allergic rhinoconjunctivitis did not find differences in the combined medication score or pollen sIgG1 and sIgG4 (240). Recombinant allergen hybrids help to reduce the administered dose, long-term immunogenicity, and treatment duration, but late-phase reactions are still seen due to the preservation of T-cell epitopes. Preclinical evaluation for application in AIT needs further study.

### Nanoparticles and Virus-Like Particles

Using nanoparticles and virus-like particles, allergens can be delivered so as to activate the innate and adaptive immune responses. Nanoparticles (<100 nm in size) such as liposomes, polyamides, polysaccharides, and polyesters, and virus-like particles can be used to encapsulate allergens to protect them from IgE-binding, direct covalent conjugation, or adsorption, and they are then delivered to APCs (241). Encapsulation is preferred for the mucosal and oral routes. Nanomedical platforms have the potential for achieving effective permeation in the cases of epicutaneous and intranasal delivery, and for their ability to form a depot, protect against enzymatic degradation and stimulate allergen-specific tolerance (242). *In vitro* data have shown promotion of Th1 stimulation and enhancement of maturation of APCs without any Th2 response. Patients undergoing HDM SCIT in which the allergen was encapsulated in viral particles showed 50% improvement in the medical and symptom scores compared with adjuvant alone (243).

### Nucleic Acid-Based Vaccines

Deoxyribonucleic acid and mRNA encoding the desired allergen are inserted into a bacterial plasmid. The plasmid contains non-methylated CpGs so that it can stimulate an acquired immune response. When the shot is injected, the gene contained in the plasmids is delivered to the APCs of the host. Animal models have shown immunomodulatory effects by driving Th1 induction of IFN-γ and IgG2a antibodies and suppressing Th2 sensitization (244). These vaccines are aimed at reducing severe systemic effects of AIT. mRNA vaccines are safer than DNA vaccines because foreign sequences in the DNA may fuse into a genome of a patient. Most studies had been conducted in murine models. In a study in humans, a CryJ2-LAMP plasmid vaccine administered to Japanese red cedar atopic subjects appeared to be a safe and effective treatment (245).

### T- and B-Cell Peptides

Synthetic allergen peptides containing T-cell epitopes do not activate IgE antibodies but induce T-cell tolerance. A clinical study of HDM and ragweed and grass-pollen allergies demonstrated some benefits and safety. On the other hand, increased nasal and bronchial symptoms were found in cat-allergic patients (246). B-cell peptides aim to establish protective humoral antibodies that are independent of IgE antibodies. For the development of recombinant hypoallergenic allergen, B-cell peptides that do not react with IgE antibodies are conjugated with a carrier to be used for AIT with the goal to make a safer, resulting in the generation of protective allergen-specific IgG antibodies without stimulating IgE antibody production which can block the interaction between patients IgE and natural allergen (247).

### Allergoids

The term “allergoid” refers to an allergen that was chemically modified by substances such as glutaraldehyde or formaldehyde but retains the ability to elicit an immunological response. The modification results in less-reactive B-cell epitopes by reducing IgE-binding but leaves T–cell epitopes unaltered. Allergoids thus show decreased allergenicity while improving immunogenicity. Allergoids are used primarily in allergic patients undergoing AIT. The dose-escalation phase of conventional AIT lasts up to 6 months, whereas when using allergoids, up-dosing is significantly shortened to only 4–8 weeks (248). Efficacy of allergoids has been shown for HDM, birch pollen, and grass pollen. In a real-life study from Germany, patients who underwent allergoid SCIT had significantly fewer AR and asthma symptoms than the non-AIT control group after 6 years of follow-up (249). Another study showed an increase in sIgG4 antibodies in the allergoid treatment group that was about 1.4–2.8 fold above the baseline (250). Grass-pollen allergoid also showed efficacy for nasal symptoms in the first pollen season, persisting until the third season. There was no difference in basophil activation between the allergoid and standard grass-allergen extract. Of note, immunogenicity was significantly lower in the allergoid group than in the control group (251). Allergoids have been demonstrated to be more cost-effective than and preferable to other AIT options. However, we have a poor understanding of the mechanism of action with different allergoids, and the chemical modification method has not been standardized. SCIT with allergoids appears to be efficacious and more cost-effective and provides benefits that persist for at least 1 year after cessation of AIT.

## AIT in the COVID-19 Pandemic

The COVID-19 pandemic has taken an extreme human toll, and the economic and social impacts of the pandemic are being felt globally. COVID-19 is caused by severe acute respiratory syndrome coronavirus 2 (SARS-CoV-2). Co-morbidities such as obesity, hypertension disease, chronic obstructive pulmonary disease, and cardiovascular disease are associated with severe COVID-19 (252), but AR is not a risk factor for severe disease. Currently, no immunologic or clinical evidence is available on how AIT and SARS-CoV-2 interact (253). SCIT and SLIT should be continued as long as there is no contraindication. SCIT can be an option for patients who wish to start AIT and in clinics where social distancing can be practiced (253). Confirmed COVID-19 cases should discontinue AIT, whether SCIT or SLIT, independent of disease severity until the symptoms have completely resolved and/or adequate quarantine has been put in place (254). After patients have recovered from COVID-19 and are asymptomatic, AIT can be started up again as scheduled. SLIT offers the option of self-treatment at home, thus avoiding the need to travel to or stay in an allergy clinic or hospital. Data are needed regarding patients switching from SCIT to SLIT during maintenance–phase AIT.

## Conclusion

Allergen-specific immunotherapy has been recommended in practice to treat severe AR patients who do not respond to conventional drug treatments. AIT induces allergic immune tolerance by enhancing various regulatory cells to control type 2 inflammation. AIT has been shown to be effective in alleviating allergic symptoms, reducing medication requirements, decreasing allergen reactivity, improving the quality of life, and preventing the development of asthma. However, conventional SCIT has disadvantages of requiring numerous injections and visits to the clinic, high cost, and systemic allergic reactions. Multiple administration routes for AIT provide alternatives and help to improve patient compliance and safety. New biologicals and advanced technologies are being developed to further improve the effectiveness of AIT.

## Author Contributions

All authors listed have made a substantial, direct and intellectual contribution to the work, and approved it for publication.

## Conflict of Interest

The authors declare that the research was conducted in the absence of any commercial or financial relationships that could be construed as a potential conflict of interest.

## Publisher's Note

All claims expressed in this article are solely those of the authors and do not necessarily represent those of their affiliated organizations, or those of the publisher, the editors and the reviewers. Any product that may be evaluated in this article, or claim that may be made by its manufacturer, is not guaranteed or endorsed by the publisher.

## Abbreviations

AAMs: alternatively–activated macrophages
AIT: Allergen-specific immunotherapy
APCs: Antigen-presenting cells
AR: Allergic rhinitis
Bregs: Regulatory B cells
CCL: C-C Motif Chemokine Ligand
DCregs: Regulatory dendritic cells
DCs: Dendritic cells
EPIT: Epicutaneous immunotherapy
FAB: Facilitated allergen binding
HDM: House dust mite
IFN-γ: Interferon-γ
Ig: Immunoglobulin
IL: Interleukin
ILCs: Innate lymphoid cells
ILIT: Intra-lymphatic immunotherapy
NK: Natural killer
PBMCs: Peripheral blood mononuclear cells
PGD2: Prostaglandin D2
SCIT: Subcutaneous immunotherapy
SLIT: Sublingual immunotherapy
TGF-β: Transforming growth factor-β
Tfh: Follicular helper T
Th: T helper
Tregs: Regulatory T cells
TSLP: Thymic stromal lymphopoietin.
